# CMR validation of left ventricular volumes and ejection fraction measured by the IQ-SPECT system in patients with small heart size

**DOI:** 10.1186/s13550-023-00987-2

**Published:** 2023-04-24

**Authors:** Hua Wei, Jiaojiao Wu, Ke Han, Guang Hu, Hongliang Wang, Xiaoshan Guo, Haiyan Liu, Zhifang Wu, Sijin Li

**Affiliations:** 1 Department of Nuclear Medicine, First Hospital of Shanxi Medical University, Taiyuan, 030001 Shanxi People’s Republic of China; 2 Collaborative Innovation Center for Molecular Imaging of Precision Medicine, Shanxi Medical University, Taiyuan, 030001 Shanxi People’s Republic of China; 3 Center for Post-Doctoral Studies, Second Hospital of Shanxi Medical University, Taiyuan, 030001 Shanxi People’s Republic of China

**Keywords:** IQ-SPECT, CMR, Small heart, Left ventricular ejection fraction, Reconstruction parameters

## Abstract

**Background:**

The IQ-SPECT system is equipped with multifocal collimators and uses ordered-subset conjugate gradient minimization (OSCGM) as its reconstruction algorithm, achieving a shorter acquisition time than conventional SPECT. Left ventricular ejection fraction (LVEF) is overestimated by conventional SPECT in patients with small heart size. In this study, we compared IQ-SPECT with conventional SPECT and cardiovascular magnetic resonance (CMR) for the estimation of LVEF in patients with small hearts (males: EDV ≤ 60 ml, ESV ≤ 25 ml; females: EDV ≤ 45 ml, ESV ≤ 20 ml).

**Methods:**

The study consisted of 49 consecutive patients (20 normal and 29 with small heart size) undergoing gated myocardial perfusion imaging (GMPI) with a 99mTc-labelled agent during stress or rest to assess the risk of coronary artery disease (CAD). The data were reconstructed using filtered back-projection (FBP) for conventional SPECT and OSCGM for IQ-SPECT. ESV, EDV, and LVEF were calculated using quantitative gated SPECT (QGS). To determine the optimal ordered-subset reconstruction parameters, we compared the LVEF from SPECT to the corresponding measurement from CMR.

**Results:**

EDV, ESV, and LVEF values obtained from IQ-SPECT and conventional SPECT showed that the results of these two forms of SPECT were significantly correlated, although the EDV and ESV obtained by IQ-SPECT were higher than those obtained by conventional SPECT. IQ-SPECT yielded lower LVEF measurements than conventional SPECT (normal heart size: 50.6 ± 4.3% vs. 73.4 ± 8.4%, P = 0.002; small heart size: 62.1 ± 7.8% vs. 75.0 ± 11.4%, P < 0.001). There were no significant differences in LVEF measurements made by IQ-SPECT and CMR (normal heart size: 50.6 ± 4.3% vs. 53.2 ± 5.8%, P > 0.05; small heart size: 62.1 ± 7.8% vs. 64.6 ± 8.8%, P > 0.05). Five subsets (S) and 12 iterations (I) did not differ significantly in LVEF between CMR and IQ-SPECT for patients with small hearts (64.6 ± 8.8% vs. 62.1 ± 7.8%, P = 0.120), while 3 S and 10 I were the best parameters for patients with normal heart size (50.6 ± 4.3% vs. 53.1 ± 5.8%, P = 0.117).

**Conclusion:**

With CMR as the standard, IQ-SPECT yields more reliable LVEF values than conventional SPECT for populations with small heart size. The best reconstruction parameters from IQ-SPECT were 5 S and 12 I for patients with small hearts.

## Background

The IQ single-photon emission computed tomography (IQ-SPECT) system is a new instrument for imaging myocardial perfusion. It is equipped with a dedicated Smart-Zoom collimator for examination of the heart [1]. The advantages of IQ-SPECT over conventional SPECT include high sensitivity, high spatial resolution, short scan time, a low dose of injection imaging agent, and low doses of radiation [2–6]. The outlook for its clinical application is broad. Using coronary angiography as a gold standard, Ogino et al. [7] demonstrated that the diagnostic sensitivity, specificity and accuracy of IQ-SPECT for the treatment of coronary artery disease (CAD) were 85%, 83%, and 84%, respectively. Matsutomo et al. [8] demonstrated a significant correlation between IQ-SPECT and conventional SPECT examination findings in terms of the level of uptake of radioactivity from the left ventricular (LV) wall and the LVEF.

SPECT examination of gated myocardial perfusion, which is used to evaluate LVEF, is not accurate in patients with small hearts [9–13]. A number of studies have reported that the LVEF is measured more accurate by the IQ-SPECT system than by conventional SPECT systems with high resolution and low energy [3–5, 8]. Nevertheless, only a few IQ-SPECT-related studies have focused on patients with a very small LV volume. Yoneyama et al. [6] found that, by quantitative gated SPECT (QGS) calculation, there was a significant difference in LVEF as measured by conventional SPECT and by IQ-SPECT (75.0 ± 9.6% vs. 79.5 ± 8.3%, P = 0.001), whereas the LVEF calculated using the cardioREPO software was not different (72.3 ± 9.0% vs. 74.3 ± 8.3%) in patients with small heart size, which was commonly seen in Asian women [14].

For this reason, the current study used cardiovascular magnetic resonance (CMR), which is internationally accepted as the “gold standard” for determining cardiac function parameters [15, 16]. In this study, we investigated the correlations of end diastolic volume (EDV), end systole volume (ESV), and LVEF measured using IQ-SPECT, low-energy high-resolution (LEHR)-SPECT and CMR. We then optimized the parameters for ordered-subset conjugate gradient minimization (OSCGM) reconstruction using IQ-SPECT for patients with small hearts.

## Methods

### Patient population

The study comprised 49 patients who underwent IQ-SPECT resting gated myocardial perfusion imaging (GMPI) for CAD risk assessment at the Nuclear Medicine Department of our hospital from November 2018 to December 2020. The patients underwent IQ-SPECT GMPI followed by LEHR-SPECT GMPI, after which they underwent CMR examination within 1 week. The work was conducted in accordance with the Declaration of Helsinki (2000), issued by the World Medical Association. Approval for this study was obtained from our hospital (2018–002). Written informed consent was obtained from all participants.

Among the 49 patients, 29 had small hearts, and 20 had normal-sized hearts. Inclusion criteria for the small heart group, based on the cardiac function parameters from IQ-SPECT, included EDV ≤ 60 ml and ESV ≤ 25 ml for males and EDV ≤ 45 ml and ESV ≤ 20 ml for females [9, 13, 17]. The normal heart group was defined by EDV 61–120 ml and ESV > 25 ml for males and EDV 41–110 ml and ESV > 20 ml for females [14, 18, 19]. The exclusion criteria were as follows: (1) arrhythmia; (2) myocardial infarction affecting ≥ 3 of the 17 defined segments of the myocardium (myocardial infarction could result in inaccurate delineation of the myocardial contour); or (3) contraindications to CMR.

### Myocardial perfusion imaging procedure

#### Image acquisition

**Fig. 1:**
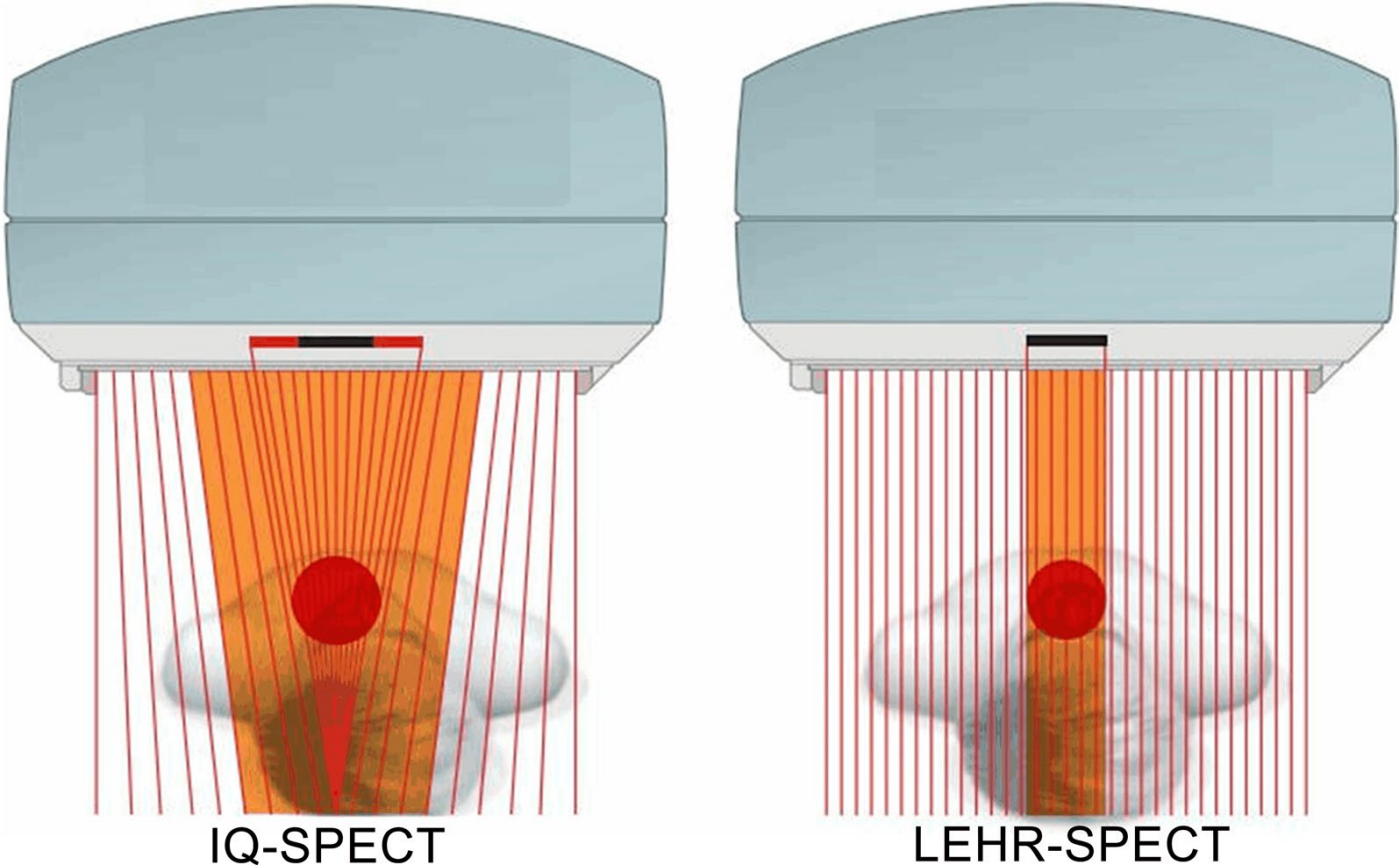
Schematic diagrams of IQ single-photon emission computed tomography (SPECT) and low-energy high-resolution (LEHR)-SPECT

Each patient was randomly assigned to undergo IQ-SPECT and LEHR-SPECT examinations in succession by two experienced nuclear medicine physicians. 99mTc-methoxyisobutyl isonitrile (MIBI) (99mTc was purchased from Atomic High-Tech Company, and MIBI was supplied by Jiangyuan Manufacturing Factory, Jiangsu Institute of Atomic Medicine) was selected as the imaging agent; this tracer has been shown to have a radiochemical purity of > 95%. A Symbia T16 dual-detector SPECT apparatus (Siemens Medical Solutions USA, Inc.) was selected as the imaging instrument. Subjects received a 20 ± 2 mCi intravenous injection of 99mTc-MIBI in a fasted state. Subjects consumed a fatty meal 15 min later and were imaged 60 min later. To acquire images, IQ-SPECT with gating technology (acquisition of 8 frames per cardiac cycle) and a SMART-ZOOM collimator (Fig. 1) was applied, with a matrix size of 128 × 128 and × 1.00 zoom. Images were acquired using a digital camera. A 20% energy window (centred on 140 keV) was used. Patients assumed the supine position with both arms holding the head in a fixed position. The detector was positioned near the chest wall. Projection datasets were acquired over a range of 208° from a right anterior obliquity of 38° to a left posterior obliquity of 66°. An acquisition rate of 25 s/frame was used, and 17 frames were acquired per detector, for a total of 34 frames. This resulted in a total acquisition time of 8 min. LHER-SPECT with gating technology (acquisition of 8 frames per cardiac cycle) and an LHER collimator (Fig. 1) was used to acquire the images, with a matrix of 64 × 64 and × 1.45 zoom. A 20% energy window (centred on 140 keV) was used. Patients were supine with both arms held with their head in a fixed position. The detector was positioned near the chest wall. Projection datasets were acquired over a range of 180° from a right anterior obliquity of 45° to a left posterior obliquity of 45°. An acquisition speed of 35 s/frame was used, and 62 frames were acquired, for a total acquisition time of approximately 20 min (Table 1).

**Table 1:** Common SPECT acquisition and reconstruction parameters

Parameters	Conventional SPECT	IQ-SPECT
Reconstruction algorithm	Filtered back-projection	OSCGM Flash 3D
Collimator	LEHR	SMARTZOOM
Energy window	140 keV ± 10%	140 keV ± 10%
Number of projections	62 views (31 per detector, 2 detectors)	34 views (17 per detector, 2 detectors)
Rotation range	180°	208°
Acquisition time (min)	20 (35 s per projection)	8 (25 s per projection)
Magnification	× 1.45	× 1.00
Rotation radius (cm)	25	28
Number of iterations		QGS 12
Number of subsets		QGS 5
Updates		20
Gaussian filter (mm)		7
Butterworth filter (Hz)	Butterworth order: 8; cut-off: 0.45	
Matrix	64 × 64	128 × 128
Pixel size (mm)	6.6	4.8

### Image processing and reconstruction

IQ-SPECT images of all subjects were reconstructed using the Siemens Flash3D iterative reconstruction algorithm with 5 subsets (S) and 12 iterations (I); the default settings for the machine were used for the reconstruction. The reconstruction parameters of the 3–10 method consisted of 3 S and 10 I; the 5–12 method used 5 S and 12 I, the 5–15 method used 5 S and 15 I, the 8–12 method used 8 S and 12 I, the 8–15 method used 8 S and 15 I, and the 8–18 method used 8 S and 18 I.

Filtered back-projection (FBP) was used for LHER-SPECT image processing, and a Butterworth filter was selected. The reconstruction parameters were set to the default parameters for the machine: cut-off frequency = 0.6 and slope steepness factor = 5. For the LV function parameters, which included EDV (ml), ESV (ml), and LVEF (%), images acquired using the different reconstruction parameters for IQ-SPECT and those acquired using LHER-SPECT were measured automatically using the software package QGS. The same procedure was used for the functional parameters.

### Cardiac magnetic resonance imaging procedure

#### Image acquisition

All participants underwent ECG-gated CMR detection within 1 week of myocardial perfusion imaging. A 3.0-T Siemens MAGNETOM Vida MRI system (Siemens, Erlangen, Germany) was used. CMR examination was performed with an eight-element phased-array cardiac coil for signal reception. Breath-hold cine CMR images in the two-chamber, four-chamber, and short-axis planes were acquired with True FISP (fast imaging with steady-state precession) bright-blood sequences to localize the heart. On the basis of the vertical and horizontal long axes of the left ventricle, the short true axis was determined, spanning the left ventricle from the base to the apex. The slice thickness was 6 mm, the slice spacing was 2 mm, the imaging field of view (FOV) was 360 mm, the acquisition time was defined as the sum of the end-diastolic and end-systolic breath-hold acquisition times, and the matrix was 256 × 256. Image acquisition was performed using ECG gating technology.

### Cardiac function analysis procedure

**Fig. 2:**
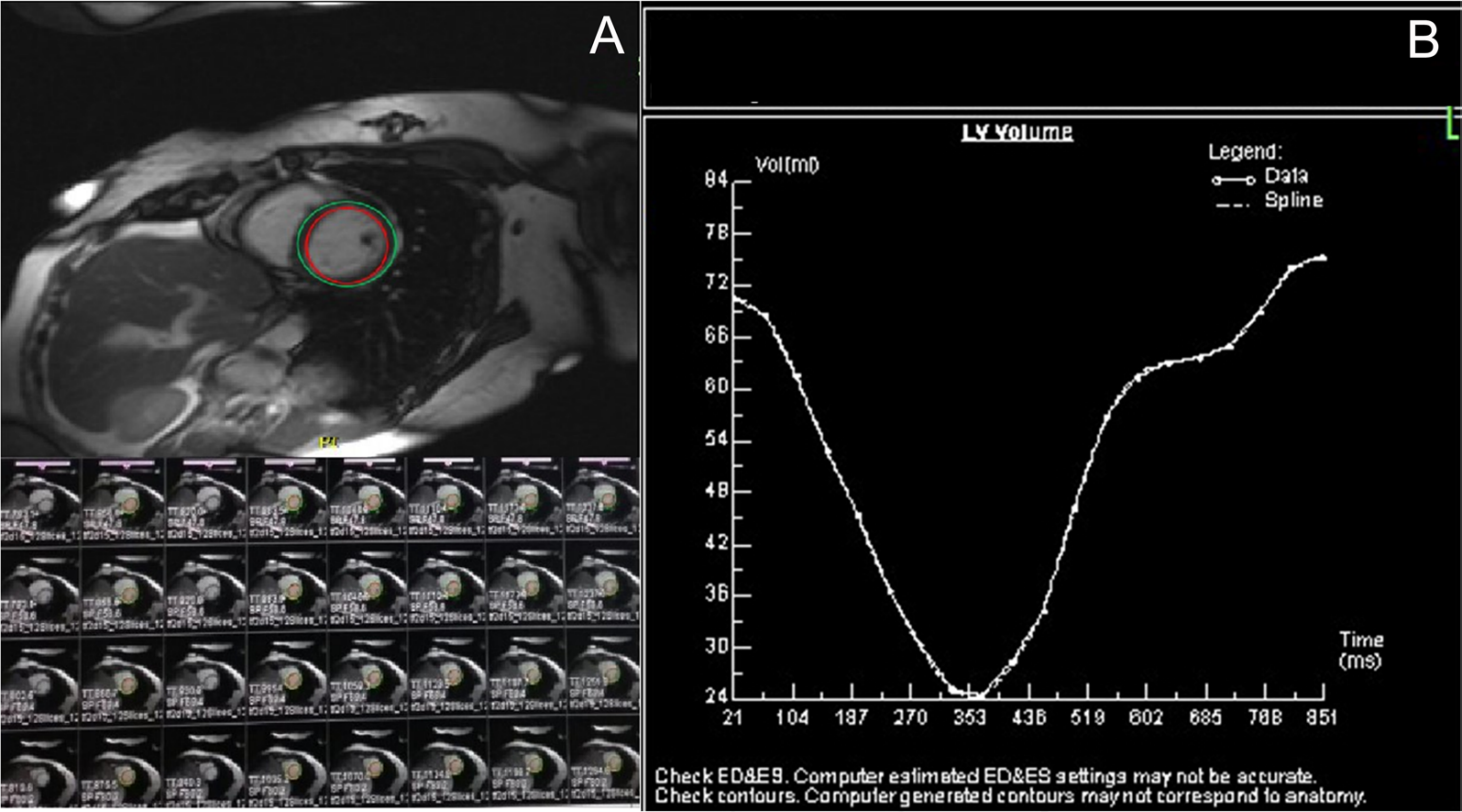
Procedure for cardiac function analysis using cardiovascular magnetic resonance (CMR)

Cardiac function was analysed and processed across the short-axis images. Syngo.via software (SIEMENS VB10B workstation) was used for image processing, and the endocardium and epicardium were delineated slice by slice from the apex to the level of the mitral valve on the short axis of the heart. Papillary muscle and chordae tendineae were included in the measurement of heart function (Fig. 2). The process of measuring cardiac function using CMR is shown in Fig. 2. To measure EDV, ESV, and LVEF, the red circle is placed in the endocardium, and the green circle is placed in the epicardium; these are delineated on a slice-by-slice basis from the apex to the level of the mitral valve on the short axis of the heart over the course of a cardiac cycle. A schematic diagram for CMR measurement of cardiac function is shown in Fig. 2.

### Statistical analysis

Statistical analyses were performed using SPSS software version 21 (IBM, Armonk, NY, USA). Continuous variables are presented as the mean ± standard deviation (SD) and were analysed using Student's t test if they followed a normal distribution according to the Shapiro‒Wilk test; otherwise, they are presented as the median (range) and were analysed using the Mann‒Whitney U test. Categorical data are presented as n (%). EDV, ESV, and LVEF determined using conventional SPECT, IQ-SPECT, and CMR were compared using Spearman correlation analysis, and the results were compared. The differences among LVEF values are shown in absolute LVEF units (% points). The Bland‒Altman test of consistency was used for coherence analysis. The mean of the differences (bias), the 95% limits of agreement (LAs), and the 95% confidence intervals (CIs) for the bias and the LAs were calculated. The ordinate of the Bland‒Altman plot was M1-M2, and the abscissa was (M1 + M2)/2, where M1 was the LVEF value measured using CMR and M2 was the LVEF value measured using different reconstruction parameters. Statistical significance was defined by a P value < 0.05.

## Results

Forty-nine consecutive patients were included in this study, 29 of whom had small hearts. Patients with small hearts were significantly older than those with normal-sized hearts (60.3 ± 9.6 vs. 44.2 ± 16.4, P < 0.001), and their height was significantly lower than that of patients with normal hearts (1.6 ± 0.1 vs. 1.7 ± 0.1, P < 0.001) (Table 2). There was a significantly higher percentage of hypertension in the small-heart group than in the normal-heart group (51.7% vs. 15.0%, P = 0.020).

**Table 2:** Characteristics of patients

Characteristic	Total	Small-heart group			Normal-heart group			P
		Male	Female	Total	Male	Female	Total	Overall small vs. overall normal
Sex (M/F, n)	49 (26/23)	12	17	29	14	6	20	0.048*
Age (years)	53.7 ± 15.0	59.3 ± 9.1	61.0 ± 10.1	60.3 ± 9.6	46.1 ± 15.3	39.7 ± 19.4	44.2 ± 16.4	< 0.001*
Height (m)	1.63 ± 0.08	1.65 ± 0.05	1.56 ± 0.05	1.60 ± 0.07	1.71 ± 0.06	1.62 ± 0.04	1.69. ± 0.07	< 0.001*
Mass (kg)	65.9 ± 10.3	67.6 ± 9.5	61.8 ± 8.5	64.2 ± 9.2	72.6 ± 11.2	58.8 ± 4.7	71.3 ± 13.9	0.162#
BMI (kg/m^2^)	24.8 ± 4.0	24.9 ± 3.4	25.4 ± 4.4	25.2 ± 3.9	24.9 ± 4.4	22.4 ± 2.2	24.9 ± 4.5	0.355#
Main risk factors (n)								
Obesity (BMI > 25 kg/m^2^)	21 (42.9%)	7 (58.3%)	7 (41.2%)	14 (48.3%)	6 (42.9)%	1 (16.7%)	7 (35.0%)	0.356^#^
Diabetes mellitus Angina pectoris	11 (22.5%)	3 (25.0%)	6 (35.3%)	9 (31.0%)	1 (7.1%)	1 (16.7%)	2 (10.0%)	0.166^#^
Angina pectoris	19 (38.8%)	5 (41.7%)	6 (35.3%)	11 (37.9%)	5 (35.7%)	3 (50.0%)	8 (40.0%)	0.884^#^
Electrocardiographic abnormalities pattern	34	6 (50.0%)	13 (76.5%)	19 (65.5%)	10 (71.4%)	5 (83.3%)	15 (75.0%)	0.479#
Coronary artery stenosis	12 (24.5%)	1 (8.3%)	3 (17.7%)	4 (13.8%)	7 (50.0%)	1 (16.7%)	8 (40.0%)	0.079#
Hypertension	18 (36.7%)	5 (41.7%)	10 (58.8%)	15 (51.7%)	3 (21.4%)	0	3 (15.0%)	0.020*

Abnormal ECG features included supraventricular or ventricular arrhythmias, ST-segment elevation or depression, T-wave elevation or inversion, right axis deviation, etc.

Data are shown as the means ± standard deviations or n (%)

The P values refer to differences between the overall small-heart group and the overall normal group. *Represents P < 0.05, and ^#^ represents P > 0.05

BMI: body mass index; ECG: electrocardiogram

### Comparison of image quality

**Fig. 3:**
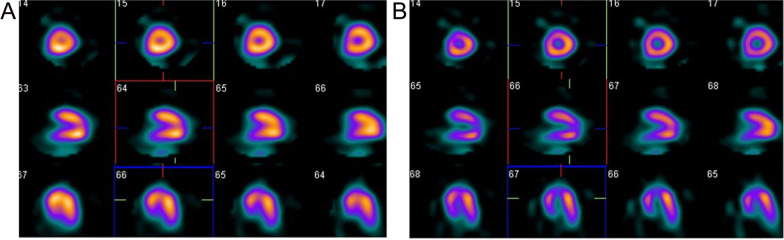
Comparison of image quality in patients with small heart size. **A** Representative tomography image of a 59-year-old female with a small heart. The number of iterations was 10, and the number of subsets was 3. **B** Representative tomography image for which the number of iterations was 18 and the number of subsets was 8. Image A is smoother than image B. The left ventricle is smaller in image A than in image B

The image was smoother when the number of equivalent iterations (subset plus iterations) was smaller, and the delineation of the endocardium and epicardium was sometimes unclear. Conversely, when the number of equivalent iterations was large, the image was sharper, and the delineation of the endocardium and epicardium was clearer. The best equivalent iteration count was 60, which outperformed higher equivalent iteration counts (Fig. 3).

### Comparison of cardiac function parameters

#### Comparison of IQ-SPECT, conventional SPECT, and CMR cardiac function parameters

EDV, ESV, and LVEF values determined using conventional SPECT and IQ-SPECT demonstrated a good correlation. There was no statistically significant difference in the value of LVEF detected by IQ-SPECT or CMR between the normal group and the small heart group (normal: 50.6 ± 4.3% vs. 53.2 ± 5.8%, P > 0.05; small heart: 62.1 ± 7.8% vs. 64.6 ± 8.8%, P > 0.05), whereas the value of LVEF detected by conventional SPECT was higher than that detected by CMR in both the group of normal subjects (73.4 ± 8.4% vs. 53.2 ± 5.8%, P = 0.002) and the group with a small heart size (75.0 ± 11.4% vs. 64.6 ± 8.8%, P = 0.007). Detection by IQ-SPECT and conventional SPECT underestimated EDV and ESV compared with CMR examination (Table 3).

**Table 3:** Comparison of EDV, ESV, and LVEF determined using conventional SPECT, IQ-SPECT, and CMR

	IQ	Conventional	CMR	Conventional vs. IQ	Conventional vs. CMR	IQ vs. CMR
(a) Normal group						
EDV (mL)	87.7 ± 14.9	63.4 ± 16.3	129.7 ± 21.5	r = 0.824 P < 0.001	r = 0.883 P < 0.001	r = 0.758 P < 0.001
ESV (mL)	43.8 ± 10.0	18.1 ± 9.2	60.7 ± 11.7	r = 0.800 P < 0.001	r = 0.638 P = 0.002	r = 0.607 P = 0.005
LVEF (%)	50.6 ± 4.3	73.4 ± 8.4	53.2 ± 5.8	r = 0.652 P = 0.002	r = 0.181 P = 0.002	r = 0.652 NS
(b) Small-heart group						
EDV (mL)	49.5 ± 9.6	45.9 ± 9.0	78.2 ± 18.8	r = 0.848 P < 0.001	r = 0.471 P = 0.010	r = 0.469 P = 0.010
ESV (mL)	19.2 ± 6.9	12.1 ± 6.8	28.1 ± 10.6	r = 0.779 P < 0.001	r = 0.539 P = 0.003	r = 0.408 P = 0.028
LVEF (%)	62.1 ± 7.8	75.0 ± 11.4	64.6 ± 8.8	r = 0.780 P < 0.001	r = 0.484 P = 0.007	r = 0.522 NS

*Conventional* conventional SPECT, *CMR* cardiovascular magnetic resonance, *IQ* IQ-SPECT, *EDV* end-diastolic volume, *ESV* end-systolic volume, *LVEF* left ventricular ejection fraction, *NS* not statistically significant

Data are represented as the mean ± standard deviation

### Comparison of cardiac function parameters obtained by IQ-SPECT using different reconstruction parameters with CMR

As the numbers of iterations and subsets increased, EDV and ESV gradually increased, and LVEF gradually decreased in the normal heart and small heart groups. The EDV, ESV, and LVEF values measured using six different reconstruction methods for GMPI were compared pairwise with those measured using CMR. There were significant differences in EDV and ESV as measured by the six GMPI methods and by CMR for the normal group. There were no significant differences in LVEF as measured by the 3–10 method, the 5–12 method, and CMR (P = 0.117 and P = 0.051). However, there were significant differences in LVEF detected by the other four methods and CMR. In the small-heart group, there were significant differences in EDV as measured by all six GMPI methods and by CMR. There were no significant differences in ESV as determined by the 8–18 method and by CMR (P = 0.054), and there were significant differences in ESV as determined by the other five methods and by CMR. No significant differences were observed in the LVEF examined by the 5–12 method and by CMR (P = 0.120), but there were significant differences in this value as determined by the other 5 methods and by CMR (Table 4).

**Table 4:** Comparison of cardiac function parameters obtained by IQ-SPECT and CMR using different reconstruction parameters

	Normal heart size	Small heart size	Normal heart size vs. CMR	Small heart size vs. CMR
3–10				
EDV	87.7 ± 14.9	46.2 ± 9.9	< 0.001*	< 0.001*
ESV	43.8 ± 10.0	14.7 ± 6.5	< 0.001*	< 0.001*
LVEF	50.6 ± 4.3	69.5 ± 8.6	0.117^#^	0.009*
5–12				
EDV	88.1 ± 14.8	49.4 ± 9.8	< 0.001*	< 0.001*
ESV	44.5 ± 10.2	19.2 ± 6.9	< 0.001*	< 0.001*
LVEF	50.0 ± 4.3	62.1 ± 7.8	0.051^#^	0.120^#^
5–15				
EDV	88.4 ± 14.9	51.1 ± 9.6	< 0.001*	< 0.001*
ESV	45.6 ± 10.4	20.9 ± 7.0	< 0.001*	< 0.001*
LVEF	49.0 ± 4.2	60.0 ± 7.1	0.013*	0.005*
8–12				
EDV	89.5 ± 15.2	52.3 ± 9.8	< 0.001*	< 0.001*
ESV	47.4 ± 10.4	22.6 ± 7.2	0.001*	0.004*
LVEF	47.4 ± 3.9	57.7 ± 7.3	< 0.001*	< 0.001*
8–15				
EDV	89.7 ± 14.8	53.1 ± 9.3	< 0.001*	< 0.001*
ESV	49.3 ± 10.6	24.1 ± 7.1	< 0.001*	0.025*
LVEF	45.7 ± 5.0	55.3 ± 6.9	< 0.001*	< 0.001*
8–18				
EDV	90.9 ± 14.4	53.8 ± 9.9	< 0.001*	< 0.001*
ESV	50.2 ± 10.7	24.8 ± 7.3	0.001*	0.054^#^
LVEF	45.1 ± 4.3	54.6 ± 6.9	< 0.001*	< 0.001*
CMR				
EDV	129.7 ± 21.5	78.2 ± 18.8		
ESV	60.7 ± 11.7	28.0 ± 10.6		
LVEF	53.2 ± 5.8	64.6 ± 8.8		

^*^Represents P < 0.05; # represents P > 0.05, indicating a statistically nonsignificant difference

*EDV* end-diastolic volume, *ESV* end-systolic volume, *LVEF* left ventricular ejection fraction, *CMR* cardiovascular magnetic resonance

**Fig. 4:**
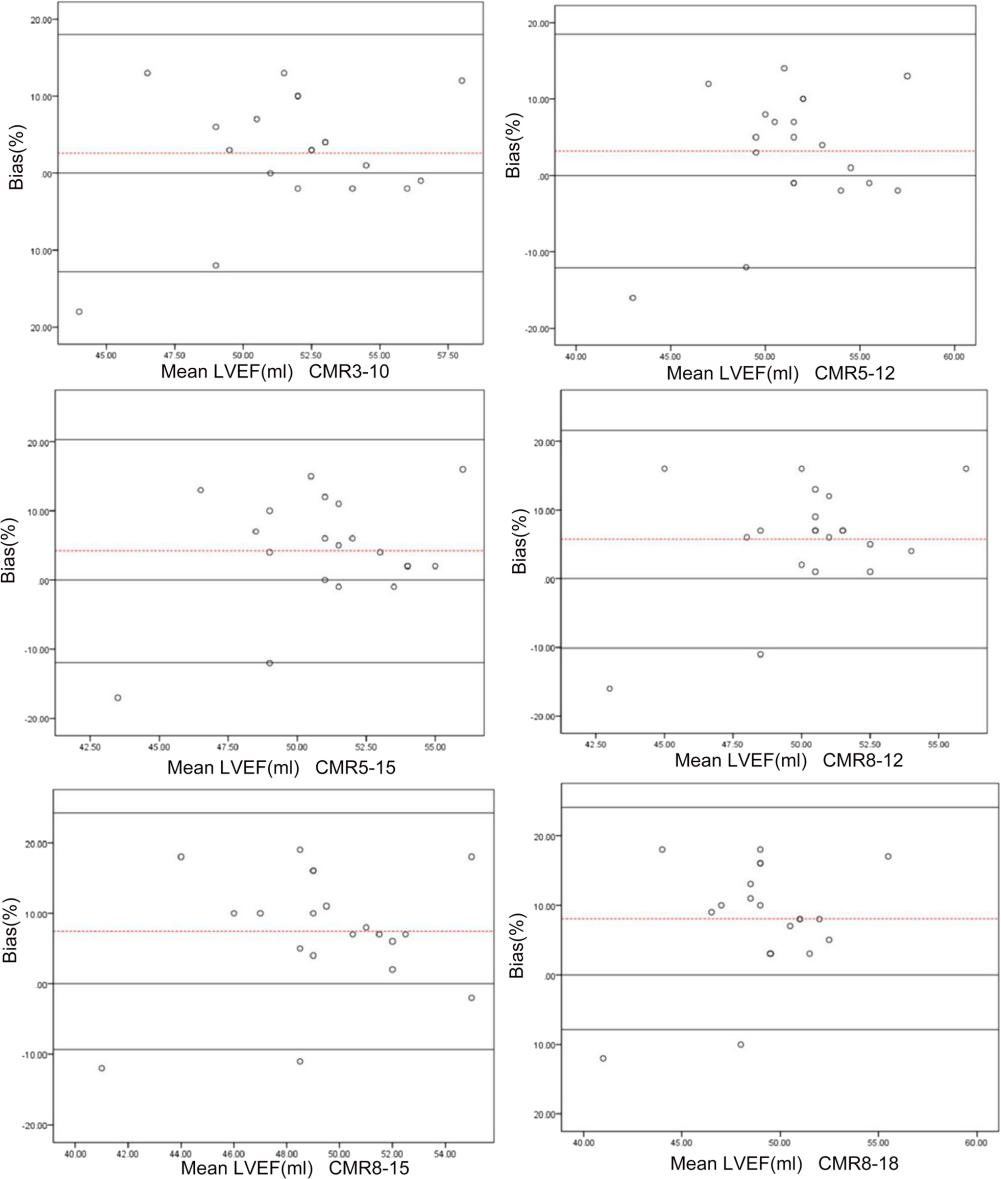
Bland‒Altman test for consistency of the left ventricular ejection fraction measured by SPECT with different reconstruction parameters and by cardiovascular magnetic resonance (CMR) in the normal group. The dashed line represents the mean of the difference (Xd). The three solid lines are the upper limit of agreement (LOA) of the difference (Xd + 1.96SD), zero, and the lower LOA of the difference (Xd − 1.96SD) from top to bottom, respectively. *Xd* mean of the difference, *SD* standard deviation of the difference

**Fig. 5:**
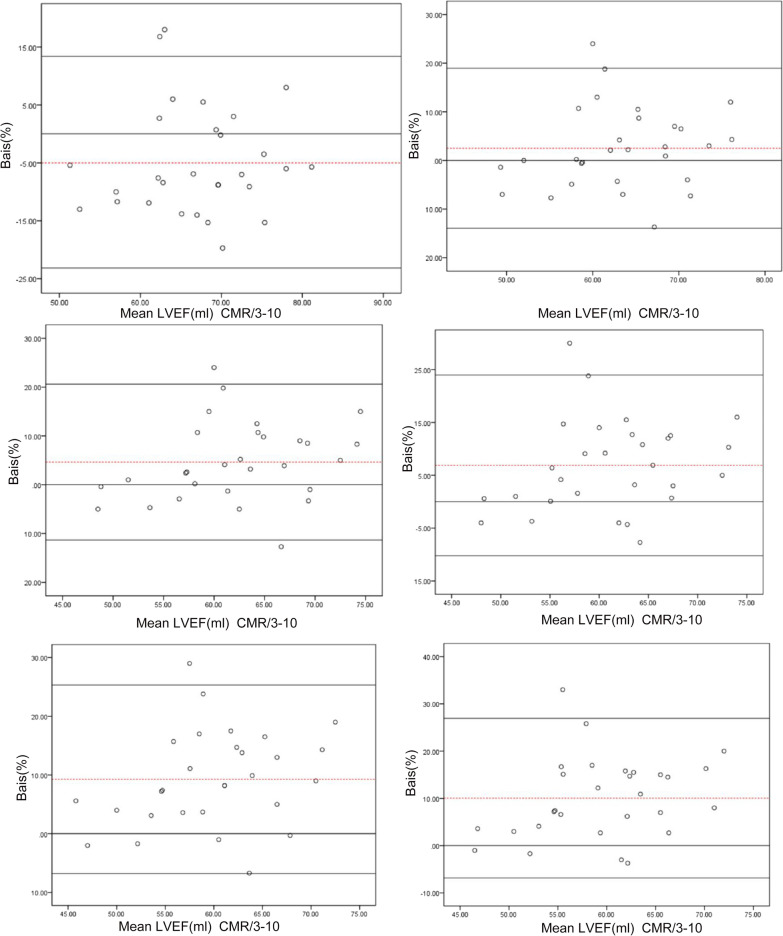
Bland‒Altman test for consistency of the left ventricular ejection fraction measured by SPECT with different reconstruction parameters and by cardiovascular magnetic resonance (CMR) in the small-heart group. The dashed line represents the mean of the difference (Xd), and the three solid lines are the upper limit of agreement (LOA) of the difference (Xd + 1.96SD), zero, and the lower LOA of the difference (Xd − 1.96SD) from top to bottom, respectively. *Xd* mean of the difference; *SD* standard deviation of the difference

Using CMR as a standard, the Bland‒Altman consistency test was used to compare the bias in the LVEF measurements made using different reconstruction parameters and CMR in the normal-heart and small-heart groups (Figs. 4, 5). In the normal-heart group, the results showed that the 3–10 method and CMR had the smallest bias, with an estimate of 2.6%. The bias produced by the 5–12 method and CMR was lowest for the small-heart group, with an estimate of only 2.5% (Table 5).

**Table 5:** Bias values and limits of agreement determined using Bland‒Altman plots

	Reconstruction parameters	Control group		Small-heart group	
		Bias/%	LOA/%	Bias/%	LOA/%
CMR	3–10	2.60	− 12.81 to 18.01	− 4.88	− 23.13 to 13.38
	5–12	3.20	− 12.07 to 18.47	2.50	− 13.96 to 18.96
	5–15	4.2	− 11.93 to 20.33	4.64	− 11.34 to 20.62
	8–12	5.75	− 10.09 to 21.59	6.88	− 10.22 to 23.98
	8–15	7.45	− 9.33 to 24.23	9.26	− 6.80 to 25.32
	8–18	8.05	− 7.92 to 24.02	10.06	− 6.83 to 26.93

*CMR* cardiovascular magnetic resonance, *LOA* limits of agreement

## Discussion

LVEF is overestimated by conventional SPECT in patients with small hearts, and the incidence of small heart size is high (nearly 80% in Asian women) [14]. On the other hand, IQ-SPECT has advantages such as fast acquisition time, high resolution, and low injection volume. Because conventional SPECT is still more common, we compared the two methods in patients with small hearts to determine which method was more similar to CMR. The objectives of this study were to evaluate EDV, ESV, and LVEF obtained using conventional SPECT and IQ-SPECT, with CMR as the gold standard, and to compare the LVEF detected using IQ-SPECT with different reconstruction parameters between those with small hearts and those with normal-sized hearts. The results suggested that IQ-SPECT might show some advantages for patients with small heart size.

CMR was selected as the reference method because it is the accepted standard for measuring global function [20]. Furthermore, volumetric accuracy was ensured by using high tissue contrast for the definition of the endocardial border [21, 22]. There are more contraindications to the use of CMR than SPECT, and there is value in comparing the LVEF detected by CMR and SPECT in patients with small hearts.

When the IQ-SPECT system was introduced in 2011, the manufacturer recommended using the Siemens Flash3D iterative reconstruction algorithm with 15 I and 2 S for the processing of gated images. In our work, we observed that the mean LVEF determined by IQ-SPECT was smaller than that determined by conventional SPECT. Hence, we compared EDV, ESV, and LVEF obtained by IQ-SPECT, conventional SPECT, and CMR. The closest agreement was observed between the LVEF detected by IQ-SPECT and that examined by CMR.

We compared 6 groups of reconstruction parameters in patients with small hearts and normal hearts to determine the effect of altering these parameters. The best Flash3D iterative reconstruction algorithm, with CMR as the standard, differed between small-heart and normal cardiac chambers. The optimal reconstruction parameters for patients with a small heart were 2 S and 15 I, and the optimal reconstruction parameters for those with normal hearts were 3 S and 10 I. In our study, there were no differences in the LVEF detected using IQ-SPECT and CMR. It is important to choose different reconstruction parameters for heart chambers of different sizes.

### Small heart size

Nakajima et al. [14] found that small heart size (EDV < 20 ml) had a prevalence of 74% in Japanese women and 13% in Japanese men. Kakhki et al. [17] also found that 85.4% of Iranian subjects had an ESV of < 25 ml (94.9% of women and 11% of men). ESV is underestimated in those with small LV volume, and LVEF is overestimated, with a greater error in females [11, 23–27]. Indeed, due to the limited spatial resolution of gamma cameras, the opposite endocardial edges of the LV overlap; thus, the ventricular cavity may become almost completely indistinct, especially at end-systole. QGS examination was shown to overestimate the ejection fraction in patients with small hearts, especially when the EDV was < 70 ml or the ESV was < 25 ml [12, 17, 23, 28, 29]. This finding implied that different thresholds must be used for subjects with normal and abnormal heart sizes.

### Comparison of LVEF detected by IQ-SPECT and conventional SPECT

The ability of gated conventional SPECT to measure LVEF, segmental wall motion, and absolute LV volumes has been extensively validated in head-to-head comparisons with clinically proven imaging methods such as planar radionuclide ventriculography, contrast ventriculography, CMR, and conventional SPECT imaging [12, 17, 23, 28, 29]. IQ-SPECT allows a significant reduction in the administered dose and acquisition time for myocardial perfusion imaging. Many studies have investigated the consistency of LVEF detected by conventional SPECT and IQ-SPECT, but the results have been inconsistent [3–6, 8, 30].

Pirich et al. [30] reported a significant difference in functional parameters derived from IQ-SPECT and conventional SPECT both after stress and during rest. The mean LVEF obtained by IQ-SPECT was reduced by 8%. The average LVEF after stress as measured by IQ-SPECT was 49.2 ± 13.0%, whereas the value obtained using conventional SPECT was 57.1 ± 12.5%. The average LVEF values as measured by IQ-SPECT and conventional SPECT during rest were 47.2 ± 12.8% and 56.4 ± 14.5%, respectively. Havel et al. [3] reported that the average LVEFs determined by IQ-SPECT and conventional SPECT were 54.1 ± 14.0% (1 S; 30 I; 14-mm full width at half maximum (FWHM) Gaussian filter) and 61.9 ± 12.2%, respectively, which were similar to our results (including all patients). Nevertheless, Yoneyama et al. [6] reported that the mean LVEF values obtained with QGS from IQ-SPECT were higher than those obtained from conventional SPECT for individuals with small and normal hearts. The average LVEF values of all patients as measured by IQ-SPECT and conventional SPECT were 68.4 ± 15.2% and 65.4 ± 13.8%, respectively. The average LVEF of patients with a small heart as measured by IQ-SPECT and conventional SPECT were 79.5 ± 8.3% and 75.0 ± 9.6%, respectively. Matsutomo et al. [8] reported that the EDV, ESV, and LVEF obtained by IQ-SPECT (1 S; 30 I; 13-mm FWHM Gaussian filter) did not significantly differ from those obtained by conventional SPECT. Although the mean IQ-SPECT-measured LVEF in that study was higher than the LVEF measured by conventional SPECT, the difference was not significant (68.3 ± 12.1% vs. 64.8 ± 11.8%, P = 0.269). Twenty-five patients were included in the study, with only seven being female, and the effect of small heart size on the IQ-SPECT system was not clarified. In the present study, we included individuals with small hearts as well as individuals with normal hearts. The results from the two groups were consistent. The mean LVEF detected by IQ-SPECT was lower than that detected by conventional SPECT for both groups.

Yoneyama et al. [6] reported EDV, ESV, and LVEF obtained using conventional SPECT, IQ-SPECT, and echocardiography. They demonstrated a good to excellent correlation between these methods. The present study found that EDV, ESV, and LVEF obtained from conventional SPECT and from IQ-SPECT showed a good to excellent correlation. In addition, we found that the LVEF measured by IQ-SPECT agreed more closely with the result of CMR detection than the LVEF measured by conventional SPECT for small hearts (62.1 ± 7.8% vs. 64.6 ± 8.8%, P = 0.120) and normal hearts (50.6 ± 4.3% vs. 53.2 ± 5.8%, P = 0.056).

### Reconstruction

The parameters of cardiac function measured using myocardial perfusion imaging are significantly influenced by the reconstruction algorithm. Nakajima et al. [14] observed that small heart size had a prevalence of 74% in Japanese women and 13% in Japanese men. In patients with small hearts, the true volume is underestimated, and this effect is greater for ESV than EDV, leading to an increase in the apparent LVEF. This small-heart effect, seen in several studies, is caused by the optimization of the SPECT reconstruction method for a myocardial wall with poor resolution [9–13, 23, 31, 32]. Nakajima et al. [23] reported that the use of a high cut-off frequency for the SPECT filter, high system resolution and proper zooming could improve gated SPECT quantification for small hearts. An improvement in spatial resolution could significantly decrease the small-heart effect.

There is currently no unified standard for optimal OSCGM parameters. Kenda et al. [33] reported that the optimal reconstruction minimization for IQ-SPECT was 1 S and 30 I. Based on these parameters, the measured LV volume was similar to the actual volume. They performed the evaluation using an RH-2 cardiac phantom (Kyoto Kagaku Co., Ltd.) containing a solution of 99mTc. Ceriani et al. [34] reported that the QGS program was able to calculate the LVEF correctly when used in conjunction with an optimized 3D OSCGM algorithm (8 S, 10 I, and an FWHM of 10 mm) but that it resulted in an underestimation of LV volumes. Duarte et al. [35] reported a more precise estimation of the quantitative parameters with OSCGM, especially with the combination of 2 I × 10 S and 2 I × 12 S. Nevertheless, they were less accurate in a validation study using a beating-heart volume phantom (ESV of 33.5 mL and EDV of 108.5 mL) than in studies of patients with small hearts. The present study found that the optimal minimization of reconstruction for IQ-SPECT was 5 S and 12 I with an FWHM of 8 mm. With these settings, SPECT LVEF was similar to that of CMR LVEF. The ESV was similar to that detected by CMR with 8 S and 18 I with an FWHM of 8 mm. The present study also showed that the optimal reconstruction minimization for IQ-SPECT was 3 S and 10 I with an FWHM of 8 mm. Based on these parameters, the LVEF for a normal heart was similar to that detected by CMR.

### Limitations

The sample size was small. Patients with large hearts were not evaluated to analyse reconstruction parameters. Another limitation was that this was a single-centre study.

## Conclusion

IQ-SPECT outperforms conventional SPECT in producing images of hearts with small ventricular chambers. The differences in LVEF as measured by IQ-SPECT and by CMR were low. IQ-SPECT and conventional SPECT measurements underestimate EDV and ESV compared to CMR exams. Different reconstruction parameters should be chosen for different heart sizes. The best reconstruction parameters of IQ-SPECT were 5 subsets and 12 iterations for patients with small heart size and 3 subsets and 10 iterations for patients with normal heart size, with the CMR measurement of LVEF as the standard.

## Data Availability

The datasets and material generated or analysed during the study are available from the corresponding author on reasonable request.

## Abbreviations

CAD: Coronary artery disease
CMR: Cardiovascular magnetic resonance
EDV: End-diastolic volume
ESV: End-systolic volume
FBP: Filtered back-projection
FWHM: Full width at half maximum
GMPI: Gated myocardial perfusion imaging
I: Iterations
LEHR: Low-energy high-resolution
LV: Left ventricle
LVEF: Left ventricular ejection fraction
MIBI: Methoxyisobutyl isonitrile
OSCGM: Ordered-subset conjugate gradient minimization
QGS: Quantitative gated SPECT
S: Subsets
SPECT: Single-photon emission computed tomography
